# Impact of iron overload on poor graft function after allo-HSCT in a patient with transfusion-dependent low-risk MDS: A case report and literature review

**DOI:** 10.1097/MD.0000000000032012

**Published:** 2022-12-23

**Authors:** Cong Wang, Munan Zhao, Yuanyuan Nie, Yan Yang, Yehui Tan, Zhonghua Du, Sujun Gao, Wei Li

**Affiliations:** a Department of Hematology in Caner Center, The First Hospital of Jilin University, Changchun, Jilin, China; b Stem Cell and Cancer Center, The First Hospital of Jilin University, Changchun, Jilin, China.

**Keywords:** allogeneic hematopoietic stem cell transplantation, iron chelation therapy, iron overload, myelodysplastic syndrome, poor graft function

## Abstract

Poor graft function (PGF) occurs in 5% to 27% of allogeneic hematopoietic stem cell transplantation (allo-HSCT) and is associated with high life-threatening complications. The etiology of PGF is complex and multifactorial, and iron overload (IOL) is considered as a predictive factor.

**Patient concern:**

A 45-years-old woman who was diagnosed as low-risk myelodysplastic syndrome in 2012 has been transfusion dependent and developed severe IOL.

**Diagnoses:**

Due to transfusion dependency and also ineffective erythropoiesis, this patient was diagnosed as IOL and developed PGF after allo-HSCT.

**Interventions:**

Deferasirox (20mg/kg/d) was administered regularly after allo-HSCT for 2 years.

**Outcomes:**

Hematopoiesis was gradually recovered during iron chelation therapy treatment after allo-HSCT and PGF was reverted.

**Lessons:**

IOL, as a prognostic factor for PGF, is a common problem in Transfusion dependent myelodysplastic syndrome patients undergoing HSCT. IOL issues should be considered at the time of diagnosis and throughout the treatment course for patients who are potential candidates for HSCT.

## 1. Introduction

Iron overload (IOL) is a common condition in patients with hematological malignancies undergoing hematopoietic stem cell transplantation(HSCT), occurring in around 30% to 60% of patients.^[1]^ IOL may have a deleterious effect on the outcomes of HSCT including HSCT toxicity, relapse risk, and even survival.^[2]^ Moreover, several pre-clinical^[3–5]^ and clinical data^[6–8]^ suggested that IOL might also affect graft function after allogeneic hematopoietic stem cell transplantation (allo-HSCT). However, the exact impact of IOL on poor graft function (PGF) and its underlying mechanism have not been fully elucidated.

This report described a case of PGF in a transfusion dependent myelodysplastic syndrome (MDS) patient receiving HSCT with severe IOL, whose condition was eventually reverted by iron-chelating therapy (ICT). In order to fully understand the association between IOL and PGF, we also made a literature review providing hematologists with an overview of current understandings of IOL in HSCT and its impact on PGF.

## 2. Case presentation

A 45-years-old woman came to our facility presenting with fatigue in May 2012. A complete blood count (CBC) indicated leukopenia (WBC count 2.7 × 10^9^/L) and severe anemia (HGB 58 g/L). Initial evaluation of reticulocyte count, folic acid, Vitamin B12, thyroid function tests, paroxysmal nocturnal hemoglobinuria and hemolytic tests all revealed negative results. Bone marrow (BM) aspiration and biopsy revealed erythroid hyperplasia and partly megaloblastoid erythropoiesis. Micromegakaryocytes were also observed. Cytogenetic test results showed a normal karyotype. An initial diagnosis of idiopathic cytopenia of undetermined significance was made, and the patient was treated with cyclosporine accompanied by supportive care, including intermittent red blood cell (RBC) transfusion therapy (1 unit/month on average).

**Figure 1.**
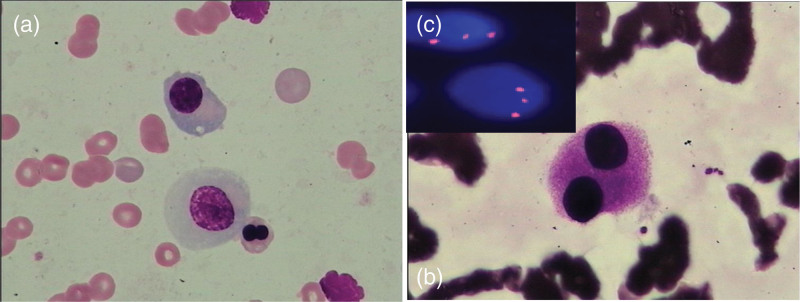
Bone marrow smear and MDS-related FISH results. (A). Bone marrow smear shows cytoplasmic hypo-granularity of granulocytes. (B). Bone marrow smear shows multinucleated erythroid cells. (C). MDS-related FISH confirms amplification of chromosome 8. MDS = myelodysplastic syndrome.

After a year of steady maintenance, the patient showed pancytopenia: WBC 2.7 × 10^9^/L, HGB 45 g/L, PLT 70 × 10^9^/L in July 2013. A BM smear revealed cytoplasmic hypogranularity of granulocytes and multinucleated erythroid cells (Fig. 1A). Approximately 5% dysplastic megakaryocytes with multiple nuclei were also observed (Fig. 1B). Cytogenetic test results revealed 47, XX, +8[2]/46, XX [9], and a MDS related FISH panel confirmed amplification of chromosome 8 in 80 of 200 interphase nuclei tested (Fig. 1C). Human leukocyte antigen (HLA)-DR15 typing was positive. The patient was then diagnosed with Unclassified MDS (IPSS: INT-1, IPSS-R: INT, WPSS: HIGH) based on the World Health Organization 2008 MDS classification. Cyclosporine and supportive care, including hematopoiesis stimulating agents as well as transfusion, were administered. At this point, the patient had developed transfusion dependence (4 units/month), and the blood cell count continued to decline progressively.

CBC test results in May 2014 showed deteriorating clinical status due to persistent pancytopenia: WBC 2.65 × 10^9^/L, HGB 58 g/L, PLT 20 × 10^9^/L. The serum ferritin (SF) level had increased to 2659 ug/L due to more frequent RBC transfusions (6 units/month). The patient was then put through ICT and was treated with deferasirox (DFX) (20 mg/kg/d) for 2 months, which eventually came to a close because of the patient’s lack of compliance. In November 2014, the patient was given a 5-day decitabine regimen (7 mg/m^2^/d) because of severe pancytopenia and a poor response to immunosuppression therapy. Bone marrow suppression after decitabine treatment lasted for as long as 40 days, and the patient developed severe infections. Two months later, the hematological values continued to deteriorate progressively: WBC 1.68 × 10^9^/L, ANC 0.54 × 10^9^/L, HGB 50 g/L, PLT 6 × 10^9^/L. BM biopsy of multiple sites showed severe hypoplasia, predominated by lymphoid cells (74%). The patient had now developed severe BM failure and showed dismal responses to current therapies. The hematological functions were primarily maintained by continuous transfusions of both RBC (8 units/month) and platelets (5 units/month). After a detailed consultation with the patient, we decided to perform allo-HSCT. High-resolution HLA genotyping revealed that the patient’s sister was HLA-compatible with her, representing an appropriate related donor. The SF level at this time had reached 16142 ug/L; therefore, during the preparatory period for HSCT, DFX (20 mg/kg/day) was administered regularly for 4 months for ICT, during which time the monthly RBC transfusion requirement remained at 7 units.

After 4 months of DFX treatment, SF levels decreased to 11849 ug/L; however, the severe pancytopenia still persisted with no signs of recovery: WBC 0.18 × 10^9^/L, ANC 0.11 × 10^9^/L, HGB 45 g/L, PLT 8 × 10^9^/L. DFX was temporarily discontinued prior to conditioning for HSCT. Busulfan-based BU/CY was chosen as the conditioning regimen. A total number of 6.958 × 10^6^/kg mobilized peripheral CD34 + cells were infused intravenously over 3 consecutive days, and cyclosporine + MMF + short term MTX were administered for graft-versus-host disease (GVHD) prevention. Neutrophil engraftment appeared at day + 21 post-transplantation, and platelet engraftment showed up at + 19. A BM smear at + 28 post-transplantation revealed active proliferation, and cytogenetic tests confirmed a normal karyotype with 100% chimerism. However, the hematological values failed to recover as expected 2 months later, as CBC declined again: WBC 1.74 × 10^9^/L, ANC 0.44 × 10^9^/L, HGB 53 g/L, PLT 9 × 10^9^/L. A BM smear revealed hypoplasia. As no signs of infection were detected, we administered a series of treatments to stimulate engraftment and hematopoietic recovery, including thrombopoietin, human gamma globulin, dexamethasone, and mesenchymal stem cell transfusion from a third-party donor. However, still no signs of recovery were seen.

**Figure 2.**
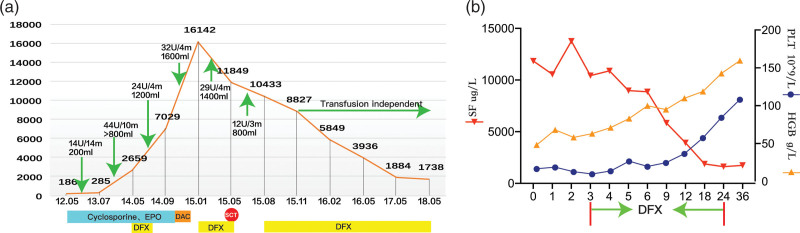
Iron overload and iron chelator DFX treatment during post-HSCT period. (A). RBC transfusion burden and SF levels, as well as treatment strategy are summarized along with disease progression. The SF level gradually increases to the peak of 16142 ug/L after transfusion of 114U RBC. B. DFX is prescribed at 3 months post-HSCT and persists for almost 2 years. The hematopoiesis is eventually recovered along with a decrease in the SF level. DFX = deferasirox, RBC = red blood cell, SF = serum ferritin.

In August 2015, 3 months after transplantation, maintenance of hematological functions still required continuous transfusions (RBC 4 units/month; platelets 1 unit/month); the SF level was 10433 ug/L at this time. Considering that the PGF might be associated with IOL, we restarted DFX at a dose of 20mg/kg/day. SF level was closely related to RBC transfusion burden and DFX treatment, which were summarized in Figure 2A. After 9-months of DFX therapy, the SF level dropped to 3936 ug/L. HGB recovered to 107 g/L, and the platelet count also increased to 33 × 10^9^/L. As shown in Figure 2B, hematopoiesis was gradually recovered during ICT treatment and PGF was reverted. By May 2017, 2 years after transplantation, the SF level had dropped to 1884 ug/L. HGB had recovered to normal levels, and PLT was maintained at 84 × 10^9^/L. At the time of last follow-up, the patient was in great condition, and the latest CBC test showed a normal result.

## 3. Discussion

Transfusion dependency and IOL are the most remarkable clinical features of this low-risk MDS patient. The level of SF gradually increased to 16142 ug/L after transfusion of 114U RBC in total (Fig. 2A). Due to transfusion dependency and also ineffective erythropoiesis of MDS, our patient developed severe IOL with extremely high SF levels. Iron overload reduction around SCT may be undertaken prior to or following SCT. Although ICT was administered for 4 months before allo-HSCT in this patient, no improvements on hematopoiesis and RBC transfusion dependency were seen. Therefore, in order to eliminate the need for transfusion and potentially cure MDS, the patient was given allo-HSCT under the circumstance of severe IOL. Unfortunately, she developed PGF 2 months after transplantation. Does IOL have a negative impact on graft function? What are the potential mechanisms? In order to provide hematologists with evidence on the understanding of IOL in HSCT and its impact on PGF, we reviewed the recent literatures which are summarized as below.

### 3.1. Association between IOL and PGF

PGF is defined as severe cytopenia of at least 2 cell lines(Hb < 10g/d, ANC < 1 × 10^9^/L, PLT < 30 × 10^9^/L) beyond day + 28 post-transplant and/or transfusion requirement in the presence of hypoplastic/aplastic BM with full donor chimerism, and in the absence of severe GVHD or relapse.^[9]^ The reported incidence of PGF varies from 5% to 27%.^[10,11]^ In this patient, considering the failure of hematological recovery after 2 months post-transplant, the achievement of full donor chimerism and adequate hematopoietic stem cells (HSCs) infusion, as well as no detectable acute GVHD or relapse, a diagnosis of PGF was made.

The etiology of PGF is complex and several factors may be related with PGF, such as donor type, ABO incompatibility, stem cell dose, intensity of conditioning regimen, GVHD, and viral infections.^[12]^ Additionally, a higher level of SF before transplantation is also one of the factors that may predict PGF.^[13]^ In a recent nested case-control study with 830 patients undergoing allo-HSCT, Gao et al^[14]^ identified 3 independent risk factors for primary PGF, including a CD34 + cell dose < 5 × 10^6^/kg, a SF level > 2000 ng/ml and splenomegaly. Moreover, Cox regression analysis in this study also suggested that PGF and high SF level were strongly associated with rapid death in these patients. IOL has rarely been reported as a cause of PGF, however, plenty of clinical studies^[7,8,15]^ showed that IOL was associated with a delay in platelet and neutrophil engraftment. And improved hematopoiesis was also observed in other studies,^[6,16]^ in which DFX was administered as ICT during the post-transplantation period. These studies indirectly reflected that IOL might be the cause of PGF, possessing adverse effects on hematopoiesis and engraftment after HSCT.

At the beginning, we administered a series of treatments to stimulate engraftment and hematopoietic recovery, however, still no signs of recovery were seen in this patient. After ICT treatment, alongside an expected decrease in SF level, we observed a progressive normalization of all cytopenias and final reversal of PGF, which in turn verified that IOL might be the main cause of PGF in this patient. Olivieri et al^[17]^ also reported a similar case of reversal of PGF with ICT after allo-HSCT for several aplastic anemia.

### 3.2. Pathologic impact of IOL on graft function

The underlying mechanism of PGF still remains unclear and the pathogenesis of PGF is probably multifactorial including immunologic issues as well as abnormalities in stem cells and the bone marrow microenvironment.^[18–20]^ Accumulating evidence have shown that increased levels of reactive oxygen species (ROS) might contribute to the occurrence of PGF after allo-HSCT by impairing both HSCs and the BM microenvironment.^[19,21,22]^ The anti-oxidant NAC can overcome the exhaustion of HSCs, improve BM endothelial cells and enhance the engraftment of HSCs by decreasing the cellular ROS levels in patients with PGF.^[23,24]^ IOL results in iron-induced toxicity mainly by ROS production which may affect hematopoietic reconstitution by impairing both hematopoietic stem cells and stem cell niche.^[25,26]^ Therefore, IOL might lead to PGF via increasing ROS levels.

IOL might impair the function of HSCs by increasing ROS production. In a mouse model,^[27]^ donor bone marrow mononuclear cells (BM-MNCs) from iron-overloaded mice were transplanted into recipient mice using and normal mice as control. Flow cytometry analysis showed that compared to those who received BM-MNCs from a normal mice, mice receiving BM-MNCs from iron-overloaded donors had lower levels of myeloid, B and T-lymphocytic lineage engraftments. In this model, IOL increased ROS production and reduced the numbers of HSCs, as well as their capacities to form colonies and engraft after transplantation. In another iron overload mouse model, Tanka et al^[26]^ demonstrated excessive iron-induced growth arrest and apoptosis in immature hematopoietic cells, which was mediated via ROS activation of p38 MAPK and JNK pathways.

Bone marrow transplantation from normal donors to IOL recipients also showed delayed hematopoietic reconstitution, which indicated that excess iron negatively impacts the hematopoietic microenvironment.^[28]^As an essential component of the BM microenvironment, epithelial cells (ECs) play critical roles in supporting hematopoiesis and thrombopoiesis.^[29]^ Using prospective case-control study,^[30]^ Kong et al found that the frequency of BM ECs was significantly reduced in PGF patients compared with good graft function patients. Activation of p38 and its downstream transcription factor was detected in BM ECs from PGF patients, characterized by increased levels of ROS and impaired proliferation, migration and angiogenesis of ECs.^[23]^ Moreover, atorvastatin, as a regulator of p38 MAPK, may be a novel therapeutic agent for PGF patients through reducing the levels of apoptosis and ROS in the BM ECs.^[31]^

### 3.3. Management of IOL around HSCT

Multiple data suggest that IOL around HSCT portend adverse clinical outcomes, including PGF, increased infection risk and relapse incidence, as well as lowered survival.^[2]^ Iron reduction therapy in patients undergoing HSCT may be beneficial for improving hematopoiesis and prolonging survival. However, the optimal strategy to reduce iron burden during different courses of HSCT, including pre-, peri- and post-HSCT, remains to be determined.

Some international guidelines^[32–34]^ recommended that MDS patients who were potential candidate to allo-HSCT should receive ICT if SF > 1000 ng/mL or transfusion burden > 20 units of RBC. A long period of regular and complete chelation therapy should be planned years before transplantation, because chelation process is really slow and gentle. However, SCT cannot be safely delayed in many patients requiring this procedure because of a risk of relapse. Therefore, the accomplishment of the reduction of IOL should not cause a delay in necessary transplantation.^[34]^ Few studies addressed IOL reduction during peri-HSCT period, and well-designed clinical trials are needed to further investigate the safety and efficacy of ICT during peri-HSCT period.

The options for IOL reduction post-SCT are serial phlebotomy for patients who have achieved reasonable hemoglobin levels, or ICT.^[35]^ DFX, an oral iron-chelating agent with possible anti-leukemia and immune modulatory effects, might be the best candidate.^[36]^ In the post-HSCT period, many studies showed a manageable efficacy and safety profile of DFX in the settings of HSCT. Improved hematopoiesis was observed in some studies which used DFX as an iron reduction agent in the post-transplantation period,^[6,16]^ which indirectly indicated that IOL might have a negative effect on hematopoietic recovery after HSCT. Moreover, Byung-Sik Cho et al^[36]^ conducted a retrospective study discussing the effect of DFX during post-HSCT period. Compared to patients not receiving ICT, DFX induced a fast decline in SF level and resulted in a better survival.

## 4. Conclusion

IOL has been identified as a predictor for PGF in clinical studies, which should arouse the attention of transplant physicians. Because IOL is a common condition in patients with hematologic malignancies undergoing HSCT, especially in transfusion-dependent MDS patients. The underlying mechanism of IOL induced PGF remains to be further investigated, and currently few studies has explored the specific toxicity of IOL on PGF. However, accumulating evidence showed that ROS plays a crucial role in the pathogenesis of PGF. As we all known, IOL results in iron-induced toxicity mainly by ROS production, which has been verified to have detrimental effects on hematopoiesis by impairing both HSCs and BM microenvironment. Therefore, we speculate that IOL probably lead to PGF via ROS producing. Actually, IOL has a series of negative impacts on HSCT endpoints. It not only induced PGF, but also increased the relapse risk and shortened survival. Therefore, IOL issues should be considered at the time of diagnosis and throughout the treatment course for patients who are potential candidates for HSCT, rather than only at the time of transplantation.

## Acknowledgments

We are grateful to the patient and his family, who gave his informed consent for publication.

## Author contributions

**Methodology:** Yehui Tan, Zhonghua Du.

**Supervision:** Yan Yang, Sujun Gao, Wei Li.

**Validation:** Sujun Gao, Wei Li.

**Writing – original draft:** Cong Wang, Yuanyuan Nie.

**Writing – review & editing:** Munan Zhao.
